# Fatal Disseminated Varicella-Zoster Virus in an Immunocompetent Woman

**DOI:** 10.7759/cureus.72855

**Published:** 2024-11-01

**Authors:** Matthew Donnan, Janakan Selvarajah, Hong Ky Ho, Annie Fung

**Affiliations:** 1 Department of Internal Medicine, Box Hill Hospital, Eastern Health, Melbourne, AUS

**Keywords:** clinical case report, encephalitis, fatal outcome, herpes zoster vaccine, immunocompetent patients, immunocompromised patient, pneumonitis, varicella zoster reactivation disseminated herpes zoster, vzv encephalitis, vzv vaccine

## Abstract

Disseminated herpes zoster (DHZ), caused by the reactivation of latent varicella-zoster virus (VZV), is usually a condition that affects the immunocompromised population. It is uncommon in a vaccinated population. A previously well, independent 91-year-old lady with no known immunosuppressing conditions presented to the hospital with a productive cough and new confusion. Several days into her admission, she developed a cutaneous maculopapular rash, and then symptoms of headache and seizures, followed by a coma. This coincided with the development of extensive neuroimaging findings, including widespread vasogenic edema and mass effect. Despite treatment with intravenous antiviral therapy, she deteriorated and passed away, with a retrospective diagnosis of DHZ. This case illustrates rapid progression of VZV infection and potential for fatality in elderly, unvaccinated but immunocompetent individuals. It provides a reminder of the importance of maintaining a degree of clinical suspicion for the diagnosis of DHZ in those with suggestive features, especially in unvaccinated individuals.

## Introduction

Varicella-zoster virus (VZV) is a common herpesvirus that causes several distinct disease entities [1]. Primary VZV infection (‘varicella’, or ‘chickenpox’) is a viral exanthem predominantly affecting children that is characterized by a febrile illness with a widespread, pruritic vesicular rash [2]. The illness is usually self-limiting and benign [1]. Following initial infection, VZV can remain latent in the neuronal dorsal root ganglia for decades [1]. Reactivation of VZV gives rise to herpes zoster (HZ) or ‘shingles’, a disease primarily of the elderly or immunocompromised [3]. HZ typically appears in a single discrete dermatome and is characterized by a ‘burning’ pain, followed by eruption of a vesicular rash [2]. Disseminated HZ (DHZ) is a potentially life-threatening complication of VZV infection and presents as a non-dermatomal rash that may be associated with visceral organ involvement, including pneumonia, encephalitis, and hepatitis [1]. DHZ can be clinically indistinguishable from primary varicella but can also occur in the absence of a rash and is known as ‘herpes sine zoster’ [2,4]. This report outlines a case of fatal DHZ in an elderly patient with no immunosuppressing conditions.

## Case presentation

A 91-year-old, functionally independent woman from home alone presented to the hospital with a four-day history of a productive cough, subjective fevers, and fluctuating mild confusion. Her past medical history was significant for an ischaemic stroke, ischaemic heart disease, atrial fibrillation, hypertension, and chronic obstructive pulmonary disease (COPD). Her regular medications included apixaban, atorvastatin, digoxin, diltiazem, esomeprazole, fluticasone-salmeterol, frusemide, spironolactone, and salbutamol as required. She was a non-smoker and drank no alcohol. She had an unclear history of primary VZV, had no history of previous HZ, and had not received vaccination against HZ. She had had no recent infectious contacts.

**Figure 1:**
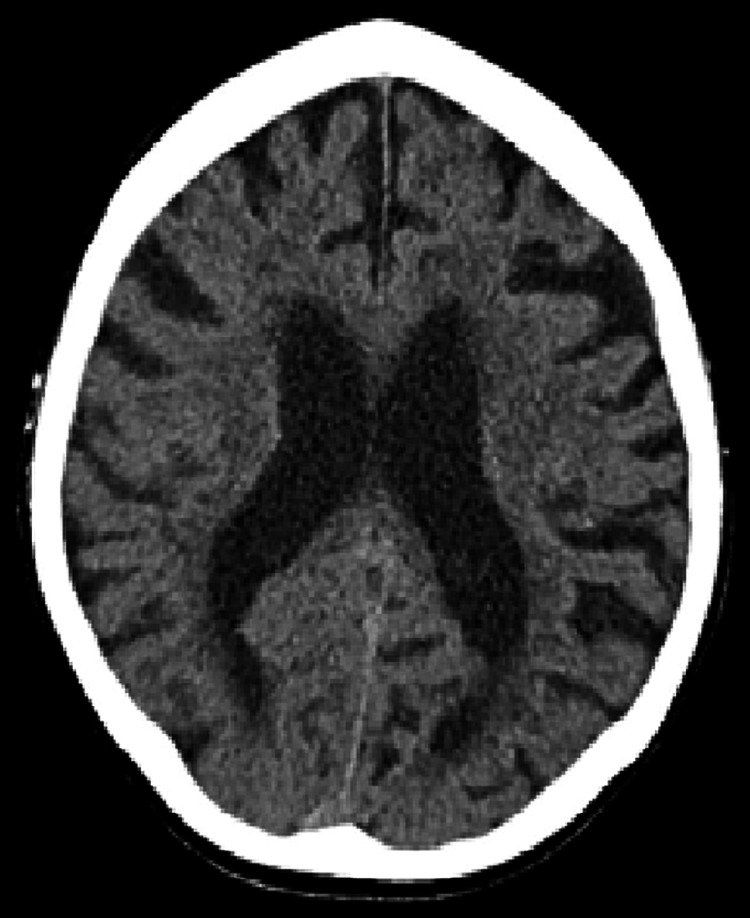
Axial CT brain on day one of admission demonstrating moderate periventricular hypodensity in keeping with chronic small vessel ischemia and age-appropriate involutional changes aside from prominence of sulci within the left hemisphere, suggestive of prior infarction.

**Figure 2:**
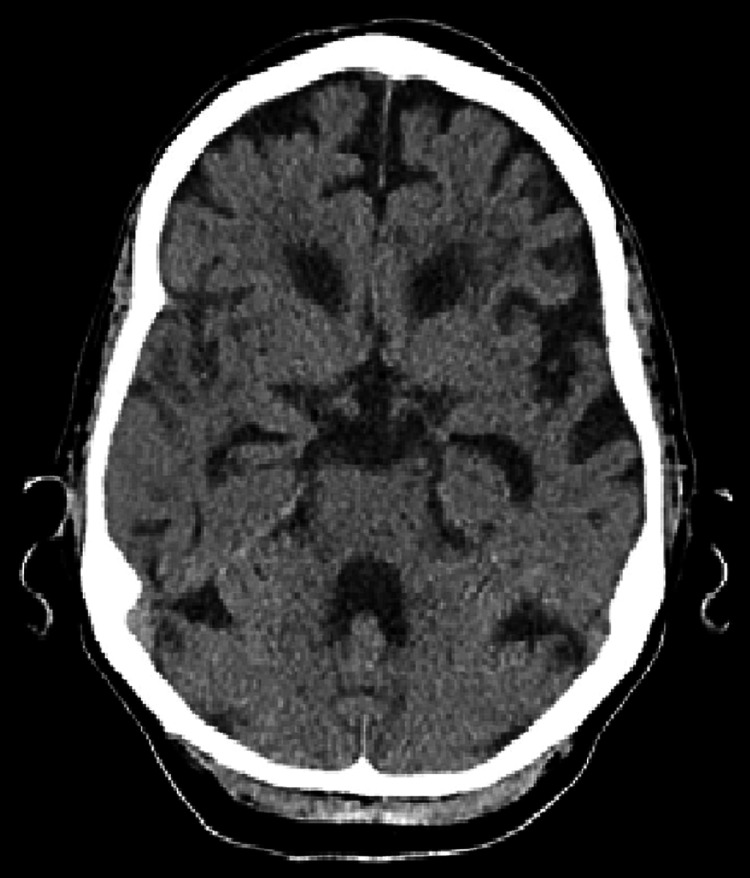
Axial CT brain on day one of admission demonstrating moderate periventricular hypodensity in keeping with chronic small vessel ischemia and age-appropriate involutional changes aside from prominence of sulci within the left hemisphere, suggestive of prior infarction.

**Figure 3:**
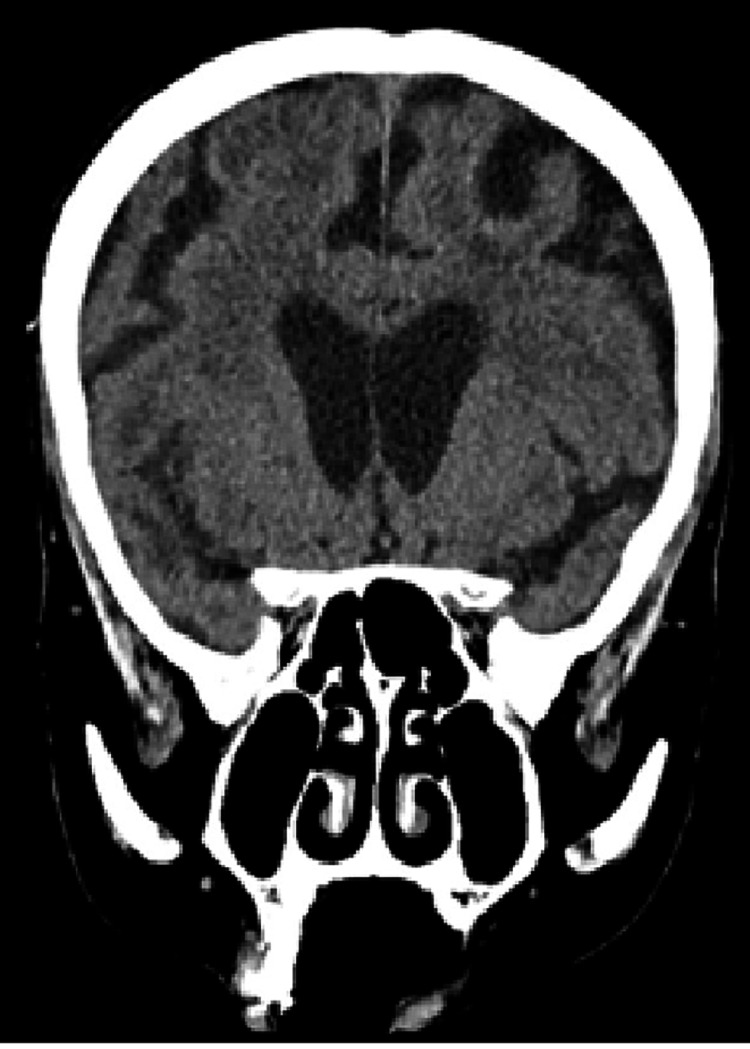
Coronal CT brain on day one of admission demonstrating moderate periventricular hypodensity in keeping with chronic small vessel ischemia and age-appropriate involutional changes aside from prominence of sulci within the left hemisphere, suggestive of prior infarction.

On presentation to the hospital, she was comfortable, haemodynamically stable, and afebrile but exhibited mild cognitive impairment. On examination, there were coarse crepitations audible at the right lung base. She was disoriented but had no focal neurological signs and no obvious rash. On presentation, her haemoglobin (Hb) was 135 g/L, white blood cell count (WBC) 11.9 x 10^9^/L, neutrophils 7.28 x 10^9^/L, and lymphocytes 3.11 x 10^9^/L. Her serum calcium, magnesium, and phosphate were within normal limits, as were her liver function and thyroid function tests. She had a slightly elevated C-reactive protein (CRP) of 8.3 mg/L. A chest X-ray (CXR) revealed mild cardiomegaly, but no gross consolidation or pleural abnormality. Admission computerized tomography scan of the brain (CT-B) demonstrated chronic small vessel ischaemic changes, as well as an area of focal left-hemisphere atrophy suggestive of prior infarction; however, it did not demonstrate any acute abnormalities (see Figures 1-3). Respiratory viral polymerase chain reaction (PCR) was negative for Influenza A, Influenza B, and respiratory syncytial virus (RSV). Blood cultures yielded no growth. A lumbar puncture was considered at presentation; however, her anticoagulation with apixaban precluded this.

**Figure 4:**
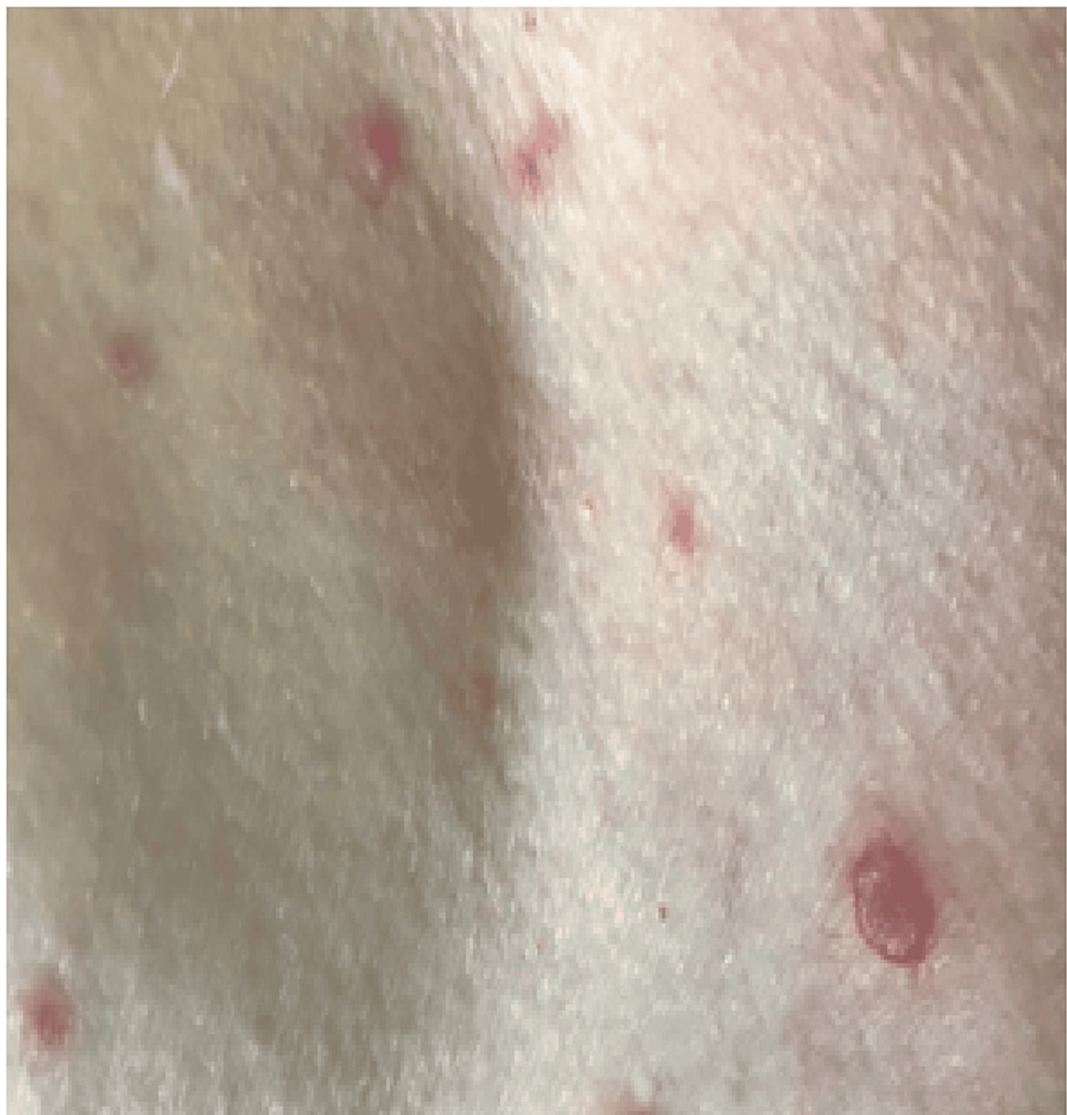
Vesicular rash on day three of admission. Distributed across the torso and upper limbs.

Following initial investigations, treatment was commenced for presumed community acquired pneumonia (CAP) with concurrent delirium. Initial treatment comprised of empirical ceftriaxone and azithromycin. In the first 24 hours of admission, she developed a persistent headache despite simple analgesia. On day two of admission, she developed a fever of 38.6°C, and a widespread, non-pruritic erythematous vesicular rash, with no discernible dermatomal pattern (see Figure 4). The lesions were 3-4 mm in size, with erythematous borders but no crusting. The patient’s WBC had risen to 19.9 x 10^9^/L, with a neutrophilia of 17.5 x 10^9^/L, and her CRP had increased to 34.7 mg/L. The vesicular rash was swabbed and sent for viral PCR, including VZV, herpes simplex virus (HSV) 1 and 2, and cytomegalovirus (CMV). HIV antigens and antibodies were negative. Following the development of the generalized rash, antibiotic therapy was changed to moxifloxacin due to concerns of an adverse drug reaction. A repeat CXR demonstrated new bilateral pulmonary infiltrates. Over the following days, the rash progressed to a centrally dusky, papulovesicular appearance with widespread distribution over the trunk, limbs, and face. This coincided with worsening cognitive impairment and somnolence. Empirical treatment for presumed viral encephalitis (intravenous acyclovir 10 mg/kg IV every eight hours) was commenced on day three of admission, within 12 hours of onset of the rash.

**Figure 5:**
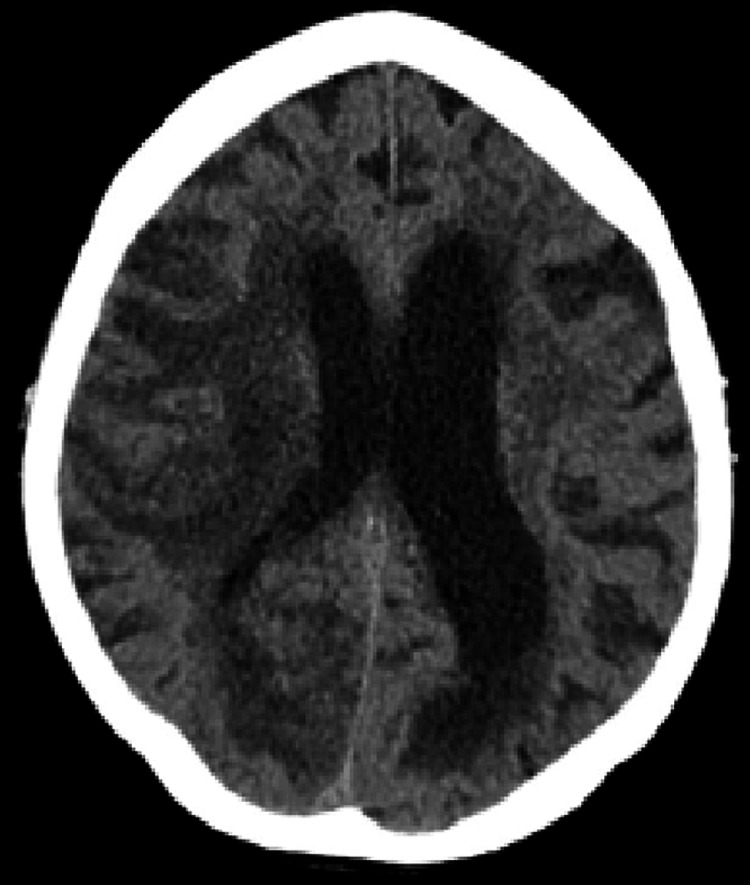
Day 4. Axial CT brain demonstrating widespread vasogenic oedema in the right frontal, temporal, and parietal lobes. Moderate mass effect with effacement of the posterior right lateral ventricle with associated midline shift. Features of early hydrocephalus with distention of the left temporal horn are also demonstrated.

**Figure 6:**
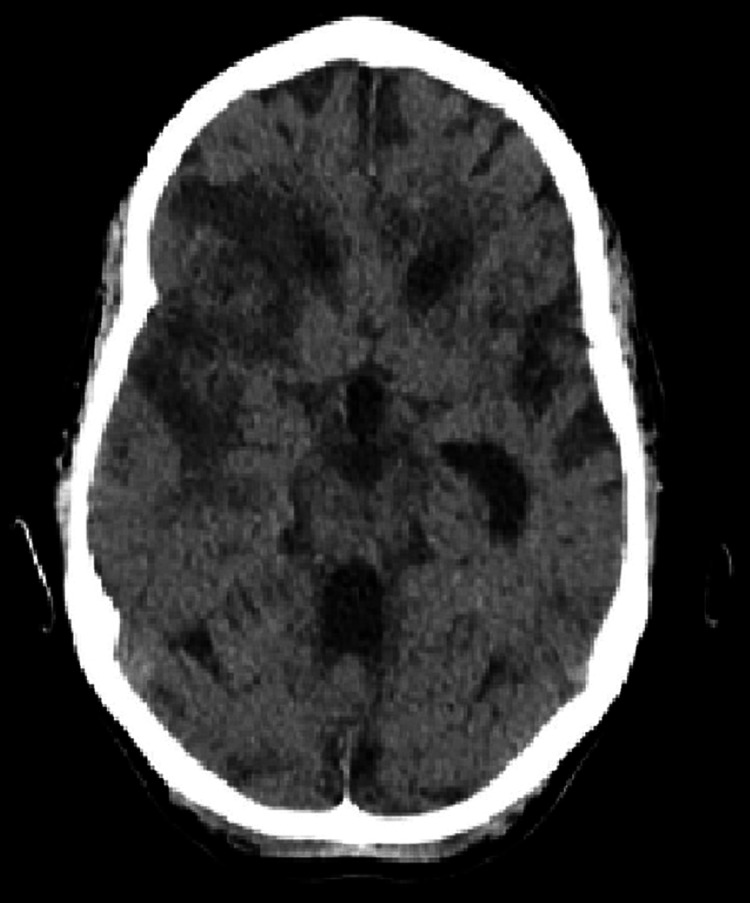
Axial CT brain on day four of admission, demonstrating a moderate mass effect with effacement of the posterior right lateral ventricle with associated midline shift.

**Figure 7:**
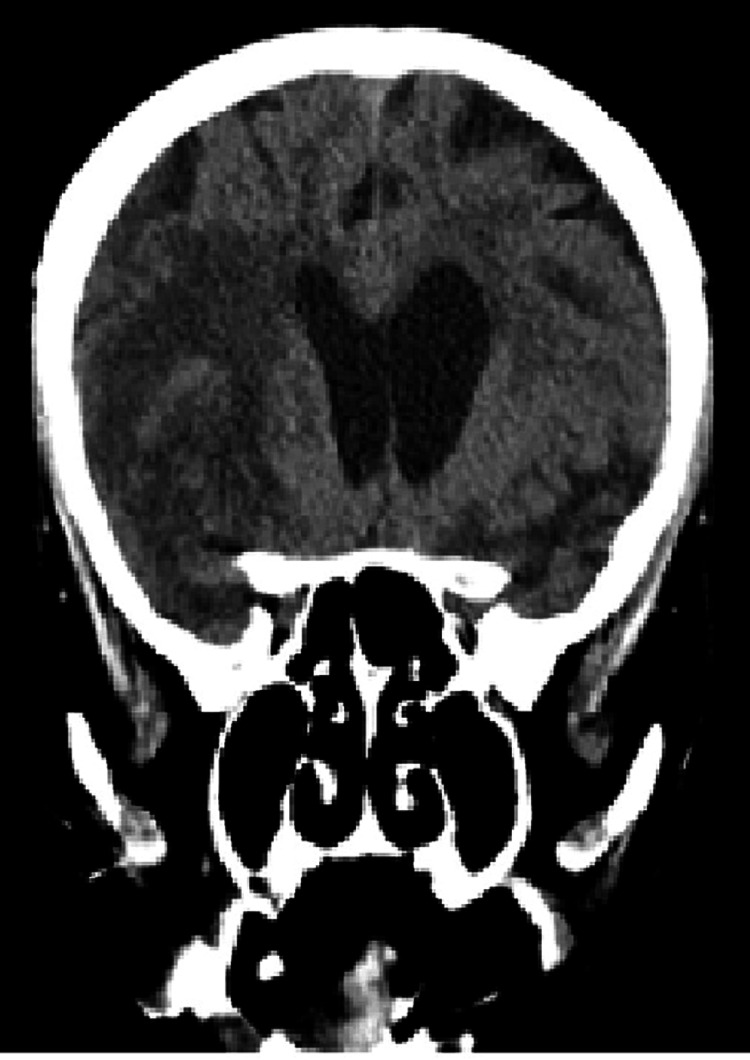
Coronal CT brain on day four of admission, demonstrating widespread vasogenic oedema in the right frontal, temporal, and parietal lobes. Moderate mass effect with effacement of the posterior right lateral ventricle with associated midline shift. Features of early hydrocephalus with distention of the left temporal horn are also demonstrated.

**Figure 8:**
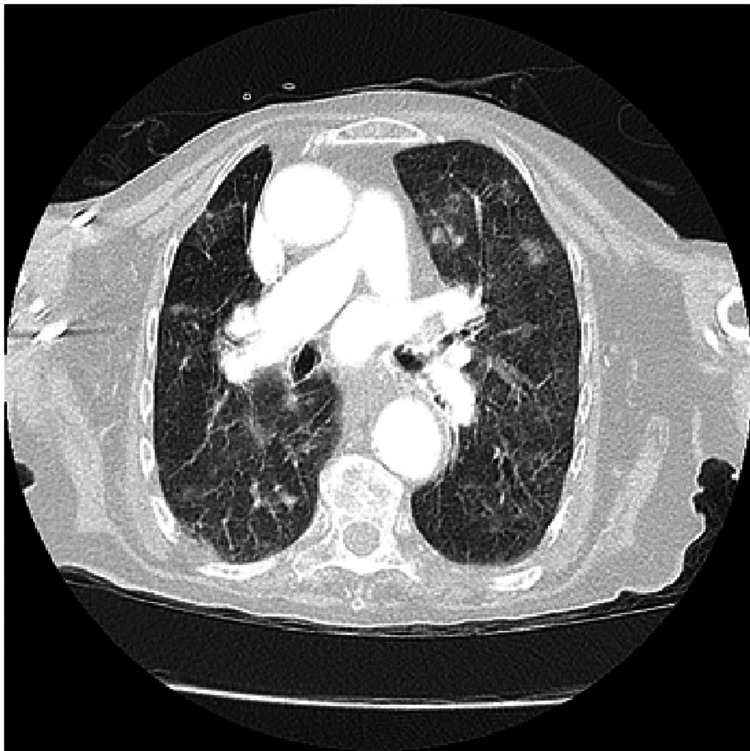
Axial CT chest on day four of admission, demonstrating widespread irregular, subcentimeter nodules with subtle ground glass peripheries suggestive of viral pneumonitis, but no frank consolidation.

**Figure 9:**
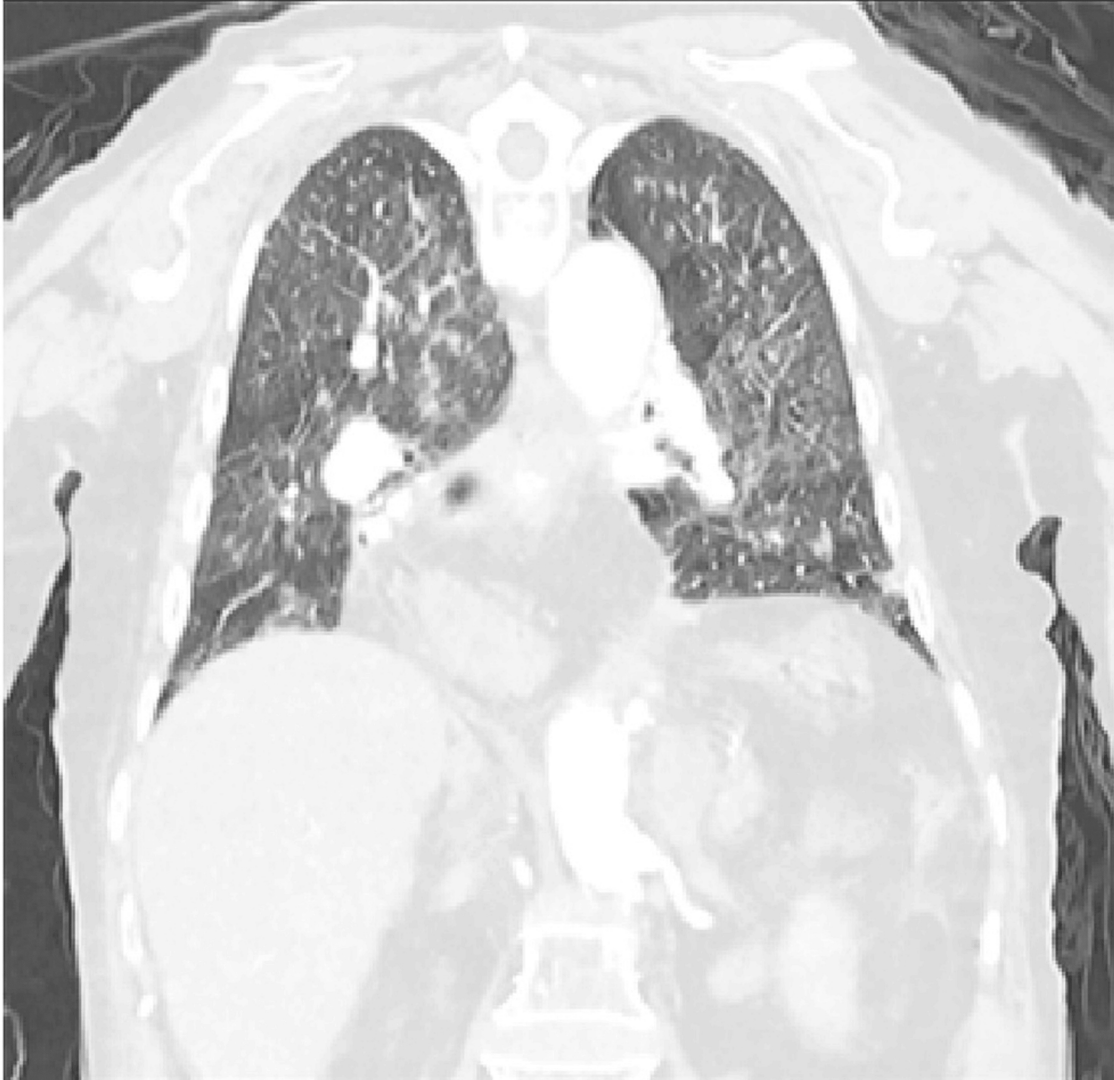
Coronal CT chest on day four of admission demonstrating widespread irregular, subcentimeter nodules with subtle ground glass peripheries suggestive of viral pneumonitis, but no frank consolidation.

A repeat CT-B on day four demonstrated new extensive widespread vasogenic oedema, predominantly affecting the right frontal, temporal, and parietal lobes associated with moderate mass effect and the development of early hydrocephalus (see Figures 5-7). This distribution of unilateral vasogenic oedema, with a pattern of acute hypodense frontal and temporal lobe changes, and periventricular involvement is characteristic of HZ encephalitis [5]. A CT chest also performed demonstrated widespread irregular, subcentimeter nodules with bilateral subtle ground glass peripheries suggestive of viral pneumonitis, but no frank consolidation (see Figures 8-9).

By day five of admission, there was a continued decline with new hypoxia requiring supplemental oxygen, and the development of seizure activity with lip-smacking, jaw clenching, rhythmic flexion, and extension of the elbows, accompanied by a reduced state of consciousness (Glasgow coma scale: 7-10). A lumbar puncture, magnetic resonance imaging (MRI) brain, and electroencephalogram (EEG) were considered but deemed unlikely to change management. She was commenced on intravenous levetiracetam and clonazepam but failed to improve with ongoing reduced levels of consciousness and oxygenation.

With continued deterioration in the patient’s condition despite anti-viral treatment and a low likelihood of meaningful recovery, the decision was made with the patient’s family to withdraw treatment and initiate palliative care. She had received 36 hours of intravenous acyclovir at the time of transition of care. The patient passed away comfortably one week after the initial presentation. 

Shortly after her death, the viral PCR of cutaneous vesicular fluid returned a positive result for VZV. Serum varicella-zoster IgG was found to be positive; however, varicella-zoster IgM was not measured, and no CSF sample was obtained during the course of this patient’s admission. Despite this, the patient met the case definition for infectious encephalitis, and in the setting of a positive viral PCR, a presumed diagnosis of DHZ complicated by VZV encephalitis and VZV pneumonitis was made [6].

## Discussion

Disseminated HZ

DHZ is an uncommon clinical occurrence among the immunocompetent population [3,7]. The annual incidence of HZ in an Australian population over 50 years old has been found to be 10.1 per 1,000 persons, increasing to 13.4 per 1,000 in those aged over 80 years. However, only 1.1% of Australian hospital admissions with HZ are with disseminated disease [8]. Complications of DHZ include pneumonitis, encephalitis, and hepatitis. These systemic complications have been reported in roughly one-third (34%) of cases of DHZ [9]. Thus, the above-reported case represents a clinical rarity. There are very few published reports of DHZ with concurrent encephalitis and pneumonitis, although Nandhagopal et al. described the case of an immunocompetent patient with non-fatal VZV encephalitis and pneumonitis who responded well to acyclovir [4].

There are many reported cases of DHZ, resulting in death in the immunocompromised population but very few in the immunocompetent population. A 2014 review of published cases of DHZ in immunocompetent patients identified only 28 patients, with no fatalities [10]. Perhaps surprisingly, it should be noted that no significant difference has been found between survival rates in immunocompetent and immunocompromised patients [9]. This may be partly explained by the fact that immunocompetent patients with DHZ are often significantly older than immunocompromised patients with DHZ, with a well-described age-related decline in immunity against VZV [9].

VZV encephalitis

VZV encephalitis predominantly affects immunocompromised patients; however, it can rarely occur in those with no known immunosuppression [11]. VZV encephalitis affects 0.1-0.2% of cases of disseminated VZV [12]. Of this number, between 2% and 20% face mortality [11,12]. This rate has been shown to be significantly higher in an immunocompromised and elderly cohort [11].

There are few cases of VZV encephalitis in an immunocompetent host described in the literature. Oh et al. presented an immunocompetent 86-year-old man with an altered mental state and dermatomal rash who was diagnosed with VZV encephalitis and successfully treated with intravenous acyclovir [13]. As with the patient described in our case, the only risk factor this patient carried was advanced age. They also identified 36 cases of VZV encephalitis reported in the medical literature. Fifteen of these patients were classically immunocompetent, none of whom died [13]. De Brouker et al., however, did describe a case series of 20 patients with VZV encephalitis, three of whom were immunocompetent patients who died [14]. All of these patients were above the age of 75.

This case represents a clinical rarity that outlines the potentially devastating effects of DHZ and emphasizes the importance of early recognition and treatment. The initial presenting complaint of this patient of fever, respiratory symptoms, or confusion is exceedingly common and can mimic other clinical entities. If not contraindicated, a lumbar puncture should be performed in the setting of unexplained confusion, fevers, and rash. This case emphasizes the importance of re-evaluation and maintaining a degree of clinical suspicion for DHZ in those with suggestive features, even if immunocompetent.

Despite having no known major immunosuppressing conditions, the patient presented in this case had multiple medical co-morbidities, including advanced age, which likely predisposed her to adverse outcomes. The incidence of VZV re-activation increases with advancing age, likely secondary to age-related loss of T-cell-mediated immunity [7,15]. Additionally, the impact of inhaled corticosteroid treatment for COPD should be considered. While there is a clear correlation between systemic corticosteroids and the risk of VZV infection, the evidence surrounding the risk of inhaled corticosteroids is less certain. One cohort study (n = 96,932) demonstrated no association between the use of ICS and the occurrence of HZ (OR: 1.00, CI: 0.94-1.07); however, a contradictory cohort study (n = 242,623) found an increased risk of HZ in those with COPD treated with long-term ICS (RR = 2.40, 95% CI: 2.20-2.60) [16-18].

In both immunocompetent and immunocompromised patients, the mainstay of treatment for DHZ is intravenous acyclovir [19]. Beyond the use of acyclovir, there is limited evidence to support other treatment options, although varicella-zoster immunoglobulin (VZIG) is routinely used for post-exposure prophylaxis of close contacts in a high-risk population [19].

This case also highlights the importance of varicella-zoster vaccination. Vaccination against VZV has been shown to significantly decrease the risk of all complications of VZV, including disseminated disease. Pooled analysis of two large vaccine trial groups demonstrated a 1.3% chance of DHZ in an unvaccinated population (n = 14, 035), with no cases of disseminated disease reported in a vaccinated population (n = 13,881) [20]. In Australia, Shingrix (a recombinant adjuvant VZV vaccine) is recommended for all those 65 years and older, all indigenous people 50 years and older, and immunocompromised people 18 years and older.

## Conclusions

This case illustrates the rapid progression and fatality of VZV infection in an elderly but otherwise immunocompetent patient despite appropriate antiviral therapy. It illustrates that systemic manifestations of VZV infection can still occur without any serious underlying immunodeficiency. Furthermore, it highlights the importance of varicella-zoster vaccination for older patients.
